# A light at the end of the tunnel – from mutation identification to a potential treatment for Alzheimer’s disease

**DOI:** 10.48101/ujms.v128.10316

**Published:** 2023-11-28

**Authors:** Lars Lannfelt

**Affiliations:** a BioArctic AB, Stockholm, Sweden; b Department of Public Health/Geriatrics, Uppsala University, Uppsala, Sweden

**Keywords:** Immunotherapy, monoclonal antibody, amyloid beta, neurodegeneration, lecanemab, Leqembi®

## Abstract

Recent advances have driven the development of immunotherapies that act by either promoting or suppressing a patient’s immune system to treat inflammation, autoimmune disease, cardiovascular disease, infectious diseases, and several cancers. In addition, research conducted over the past 25 years has identified therapeutic targets and indicated that immunotherapy could be used to treat Alzheimer’s disease (AD). Despite a number of setbacks, this approach has now led to the development of the first disease-modifying treatments for this devastating disease. A key neuropathological feature of AD is the accumulation of a ~40-amino acid peptide known as amyloid β (Aβ) in the brain and cerebrovasculature. Our detection of an Aβ precursor protein mutation that caused early-onset AD in a Swedish family by enhancing Aβ protofibril formation sharpened the focus on soluble Aβ aggregates (oligomers and protofibrils) as viable therapeutic targets. Initial studies developed and tested a mouse monoclonal antibody (mAb158) with specific conformation-dependent binding to these soluble Aβ aggregates. Treatment with mAb158 selectively reduced Aβ protofibrils in the brain and cerebrospinal fluid of a transgenic mouse model of AD. A humanized version of mAb158 (lecanemab) subsequently entered clinical trials. Based on promising Phase 2 data showing plaque clearance and reduced cognitive decline, a Phase 3 trial found that lecanemab slowed decline on the primary cognitive endpoint by 27% over 18 months and also produced positive effects on secondary clinical endpoints and key biomarkers. In July 2023, the FDA granted lecanemab a full approval, and this therapeutic antibody will be marketed as Leqembi®. This represents a significant advance for patients with AD, although many challenges remain. In particular, it is now more important than ever to identify individuals who are vulnerable to AD, so that treatment can be initiated at an early stage in the disease process.

**Figure:**
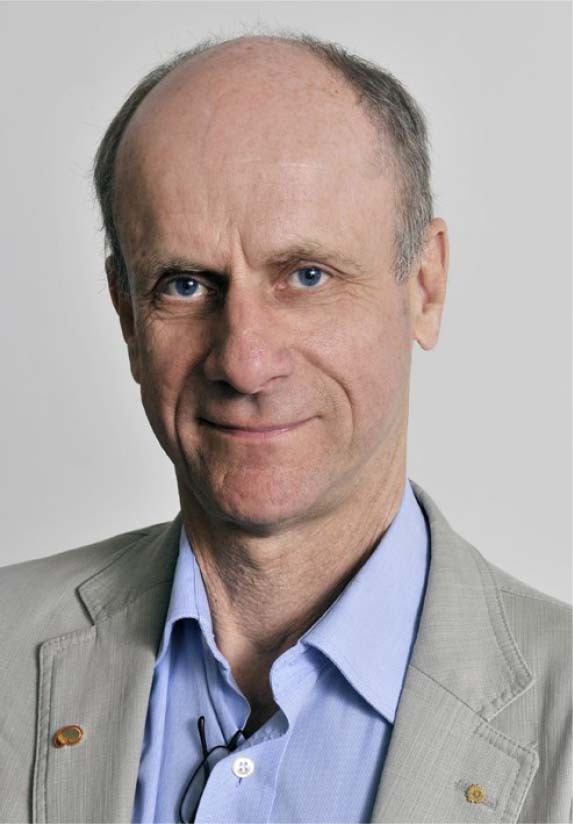
Professor Lars Lannfelt, winner of the Medical Faculty of Uppsala University Rudbeck Award 2023.

## Therapeutic antibodies

Immunotherapy provides an approach to disease treatment by either promoting or suppressing the patient’s own immune system. Antibodies are vital components of the immune system. These proteins typically consist of four polypeptides forming a tertiary structure that binds to target antigens in a highly specific manner. To employ antibodies therapeutically, patients can either be exposed to an antigen, thus induced to produce their own polyclonal antibodies (active immunotherapy) or treated with exogenous antibodies (passive immunotherapy); these can be manufactured monoclonal antibodies (mAbs) or donor-derived human polyclonal antibodies. Over the past 25 years, the use of mAbs to treat disease using passive immunotherapy has expanded rapidly in a number of therapeutic areas, including inflammation, autoimmune disease, cardiovascular disease, infectious diseases, and several cancers. Technological advances in the design, engineering, and mass-production of mAbs have driven rapid growth in the market for this class of biological drugs, estimated to have exceeded USD 180 billion in 2021 (Global Market Insights). In addition to directing the patient’s immune system toward their specific antigen, mAbs can also be coupled to other molecules such as small-molecule drugs or radionuclides and used to deliver these to a target cell, thus minimizing off-target impacts.

Active and passive approaches to immunotherapy have advantages and disadvantages. One benefit of active immunization is the capacity for a small number of vaccinations to generate a prolonged immune response. However, this approach also has some disadvantages in elderly populations due to age-related reductions in immune competency; the number of responders to immunization can be low, and the individual level of response varies. In addition, adverse effects can occur that relate to the binding epitopes of the polyclonal antibodies produced, and it is difficult to halt treatment to ameliorate these, as antibody production can persist for long periods of time. Passive immunotherapy has the advantage of allowing the reproducible administration of known doses of specific mAbs with defined antigen-binding properties, and rapid mAb clearance if side effects develop. While mAbs have proved extremely useful, the fact that they are large multimeric proteins means that they are costly to produce and need to be administered repeatedly via parenteral routes of administration.

In the context of central nervous system (CNS) disease, some antibodies may enter the brain via the lymphatic system and perivascular spaces or via a compromised blood-brain barrier (BBB). Only a small fraction (around 0.1–0.2%) of mAbs introduced into the peripheral circulation can be detected in the brain or cerebrospinal fluid (CSF) (1).

## Alzheimer’s disease

### Current understanding of disease mechanisms

In his original description of the disease, Dr. Alois Alzheimer noted the presence of abnormal structures in the brain: extracellular amyloid plaques and intracellular tangles (2). Amyloid deposition is also found in the small vessel walls of the brain, in close proximity to the BBB. This is known as cerebral amyloid angiopathy (CAA). In addition, Alzheimer’s disease (AD) is characterized by gross atrophy of the brain, extensive synapse loss, and neurochemical deficits in multiple neurotransmitter systems. Research published in 1984 led to the identification of a 40‑42-amino acid peptide known as amyloid beta or Aβ as a key component of CAA and amyloid plaques (3, 4). This peptide was found to be a break-down product of a larger protein, which was thus named Aβ precursor protein (AβPP). Subsequent research has identified hundreds of different pathogenic mutations that cause autosomal dominant early-onset familial AD; each affected family carries one of these single or double point mutations, which are either in the gene encoding AβPP or in those encoding the proteases that cleave AβPP (5). All of the pathogenic mutations affect AβPP processing, either increasing the amount or altering the type of Aβ produced. This provides support for the amyloid hypothesis of AD, which proposes that Aβ is a key initiator of the disease process and therefore an attractive target for therapeutic intervention (5). This hypothesis predicts the existence of a common pathological cascade that is driven by Aβ and eventually results in the formation of tangles composed of abnormally phosphorylated tau protein, neuronal cell and synapse loss, vascular damage, and the clinical symptoms of AD, both in individuals with familial AD and in those with no known pathogenic mutations. This latter group (sporadic AD) represents around 95% of patients, where the disease is not clearly associated with Aβ overproduction and may be more closely related to decreased clearance of the peptide (6). Furthermore, the inheritance of the apolipoprotein E4 (ApoE4) allele increases the risk for AD in a dose-dependent manner.

### Significance of Aβ aggregation state

**Figure 1:**
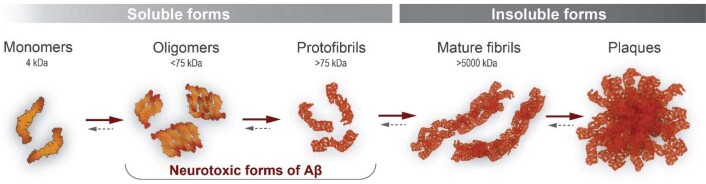
The progressive assembly of Aβ peptides results in a range of species of different sizes and levels of solubility. Aβ oligomers and protofibrils are considered to be neurotoxic. Therapeutic mAbs can be directed to Aβ epitopes that are present in one or several of these species. The aggregation process is reversible, and the presence of anti-Aβ mAbs may, therefore, shift the equilibrium between Aβ aggregate forms.

Aβ peptides exist as various species. Soluble monomeric forms, which are produced normally by most human cells, differ in length (39‑43 amino acids) due to C-terminal heterogeneity. Using synthetic Aβ peptides, these monomers have been shown to self-assemble to form Aβ aggregates of varying sizes. These range from small soluble oligomers to larger soluble protofibrils (>75 kDa) and onward to the insoluble fibrillar amyloid found in plaques (Figure 1) (7, 8). The initial assumption of the amyloid hypothesis was that fibrillar Aβ aggregates were neurotoxic. However, amyloid plaque density in the brain does not correlate with the severity of dementia (9–13), and more recent research has indicated that soluble Aβ aggregates are more neurotoxic than either the monomeric or fibrillar forms (14, 15). Large soluble oligomers have been shown to induce electrophysiological changes and neurotoxicity in rats and mice (7, 14), and Aβ42 protofibrils (but not insoluble fibrils) induce inflammation via microglial activation (16). We have therefore argued that selective removal of soluble Aβ aggregates represents an effective approach for the treatment of AD (17) (Figure 1).

In 2001, we identified the Arctic mutation within the AβPP gene sequence encoding Aβ (E693G) in a Swedish family with early-onset AD (18). Studies of Aβ with the Arctic mutation showed that the peptide had an increased propensity to form soluble Aβ protofibrils (19, 20). A useful transgenic mouse model was developed (ArcSwe). These mice, which express both the Arctic and Swedish AβPP mutations (21), display early intraneuronal Aβ accumulation and protofibril formation prior to the appearance of plaque pathology, with amyloid plaques that are as difficult to dissolve as those found in human AD (22, 23). The brain levels of Aβ protofibrils, but not of total Aβ, correlated with spatial learning performance in ArcSwe mice, supporting the idea that soluble protofibrils are neurotoxic (22). Cognitive deficits were also identified, and these occurred concomitantly with the formation of intracellular Aβ deposits, prior to plaque formation (24).

Neuroimaging of patients with the Arctic mutation using positron emission tomography (PET) with an amyloid ligand (Pittsburgh compound B) revealed that they lacked fibrillar amyloid deposits (25); this provided further evidence that protofibrils are important drivers of the disease process.

### Current small-molecule therapeutics for AD

Current AD treatment options are limited to medications that reduce dementia symptoms for 6‑12 months, without slowing the underlying neurodegenerative processes. The available drugs include acetylcholinesterase inhibitors such as donepezil and an N-methyl-D-aspartate (NMDA) receptor inhibitor (memantine). These classes of medication target the cholinergic and glutamatergic neurotransmitter changes associated with AD, respectively, without significantly prolonging the lives of affected patients. Given the rapidly aging demographic profiles of most areas of the world, new therapeutic interventions capable of slowing or perhaps even preventing disease progression are urgently needed; ideally, these treatments would even restore normal brain function.

The production, aggregation, and clearance of Aβ are all attractive targets for drug development. The β- and γ-secretase enzymes that regulate AβPP processing can be inhibited by small molecule drugs, and a number of these have advanced into clinical trials. However, they have so far failed to progress to clinical use due to either safety or efficacy issues (26). These compounds include γ-secretase inhibitors, such as tarenflurbil and semagacestat (development of both was stopped by 2010), and β-secretase inhibitors, such as verubecestat and elenbecestat, development of which has also been discontinued (27). Another interesting approach is to develop small molecules targeting aggregated Aβ (28, 29). Tramiprosate was developed by NeuroChem to bind to Aβ and reduce its aggregation and fibril formation. Progression of this compound was initially halted in 2007 following a disappointing Phase 3 trial, but subsequent longitudinal subgroup analysis of trial data indicated a potential positive effect in participants carrying two copies of the ApoE4 allele (30). A pro-drug version of tramiprosate (ALZ-801; Alzheon) is currently in a Phase 3 trial (APOLLOE4; commenced May 2021).

## Immunotherapeutic approaches to the treatment of AD

**Figure 2:**
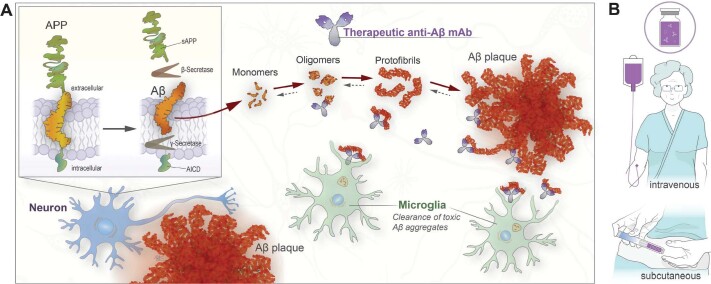
(a) Aβ is derived from the integral membrane protein β-amyloid precursor protein (AβPP) through sequential proteolytic processing by β- and γ-secretases (inset panel, top left). Additional APP cleavage products are soluble APP (sAPP) and APP intracellular domain (AICD). Aβ monomers can aggregate into toxic oligomers and protofibrils and subsequently form plaques (red), interfering with physiological function and affecting viability of neurons (blue cell). Therapeutic mAbs (purple/grey) are directed toward Aβ species of relevance to disease. mAbs binding to Aβ oligomers and/or protofibrils (red) promote clearance of these neurotoxic species, and binding to plaque deposits promotes removal though activation of microglia (green cells). (b) Anti-Aβ immunotherapy (purple vial) is administered to patients intravenously at the hospital, generally biweekly or monthly. Subcutaneous dosing of therapeutic antibodies is currently evaluated in the clinic for the treatment of AD and would offer a more convenient administration route for patients.

Immunotherapy presents a promising treatment option for AD, and there are several loci at which immunotherapies targeting AD could exert their effects (Figure 2). The presence of large amounts of anti-Aβ antibodies in the peripheral circulation can alter the equilibrium between Aβ in the blood and CNS compartments, driving passive diffusion down the resultant concentration gradient, thus promoting the clearance of monomeric Aβ from the brain. This mechanism, referred to as the ‘peripheral sink hypothesis’, has been demonstrated in animal models (31), although its applicability to AD therapeutics is unclear. Antibodies can also alter Aβ clearance by interacting with the molecules involved in ferrying Aβ into and out of the CNS across the BBB (32).

The small proportion of mAbs that do enter the CNS can also exert a variety of effects on Aβ, depending on their binding epitopes; for example, those binding to soluble forms of Aβ may increase clearance and shift equilibria between compartments, whereas those that bind to deposited fibrillar forms can trigger microglial activation to achieve plaque reduction (33). Antibodies targeting Aβ can also disrupt or promote peptide aggregation, thus altering the equilibrium between different species of Aβ or interfere with Aβ binding to other molecules and thereby reduce toxicity.

Targeting other molecules using immunotherapeutic approaches could generate mAbs that alter AβPP processing. Other strategies could involve binding to receptors on immune effectors and, thus, modulate inflammation or act within synaptic clefts to alter the cell-to-cell transport of Aβ (34, 35). Whether such approaches will prove viable for AD therapeutics remains to be determined.

Targeting molecules directly involved in the neuropathological cascade associated with AD using immunotherapy is attractive because it has the potential to reduce the risk of side effects during the long-term treatment required in this vulnerable patient population. The first attempt was launched following the striking observation that immunization of transgenic mice with fibrillar Aβ plus an immune-stimulating adjuvant resulted in anti-Aβ antibody-mediated clearance of existing amyloid deposits and the prevention of new plaque formation (36). AN1792 (Elan/Janssen/Pfizer) was subsequently developed as an active immunotherapeutic agent comprising a synthetic full-length Aβ42 peptide and an adjuvant. The progression of AN1792 was halted during a Phase 2 trial because of a serious adverse event (aseptic T-cell-mediated meningoencephalitis) in 6% of the treated patients (37). This resulted from the stimulation of a pro-inflammatory T helper (Th) 1 immune response, a finding that led subsequent vaccine developers to focus on generating immune responses that are purely humoral or involve Th2 stimulation. In addition, only ~20% of those vaccinated in this study produced antibody titers above the pre-determined therapeutic cut-off level. Nevertheless, a study conducted around 5 years after the immunizations in the original trial study found a significant reduction in the cognitive decline among the antibody responders, as compared to placebo-treated patients (38), indicating that Aβ immunotherapy could have long-term beneficial effects and providing an important proof of concept for this approach to AD treatment. Several other active immunotherapeutic agents have since entered Phase 1 and Phase 2 clinical trials. However, it is difficult to target a particular Aβ species using this approach, and the main focus of the past decade has, therefore, been to develop passive immunotherapies using standardized doses of mAbs directed to specific forms of Aβ. Many of these mAbs have failed to achieve the required endpoints during stages of the drug development process prior to Phase 3 trials. A number of potential immunotherapies (described later) have reached Phase 3 trials, and two of these have recently entered the approval process for clinical use.

Two of the earliest mAbs to be developed for AD were bapineuzumab and solanezumab. Bapineuzumab (Elan/Wyeth/Janssen/Pfizer) is a humanized murine mAb directed against the N-terminal region of Aβ; this mAb binds soluble and fibrillar Aβ. A Phase 2 PET study using amyloid PET identified a reduced amyloid load in the brains of patients treated with bapineuzumab, as compared with controls (39). Interestingly, bapineuzumab treatment was also associated with small but significant reductions in the CSF levels of total tau and of hyperphosphorylated tau (40), although no differences in CSF Aβ levels were observed. Magnetic resonance imaging of some patients involved in the clinical trials of bapineuzumab identified abnormalities indicative of cerebral microhemorrhage and vasogenic edema; this adverse event was named amyloid-related imaging abnormalities (ARIA) with microhemorrhage (ARIA-H) and/or edema (ARIA-E). The adverse event profile resulted in a dose reduction for Phase 3 trials, and the desired clinical endpoint was not achieved, resulting in discontinuation of bapineuzumab development in 2012 (41, 42).

Solanezumab (Eli Lilly) is a humanized mAb that shows selectivity for soluble monomeric Aβ, rather than targeting fibrillar Aβ. This mAb preferentially binds the mid-region of monomeric Aβ. A Phase 2 study of solanezumab in mild-to-moderate AD found a dose-dependent increase in CSF Aβ42 levels, indicating that the mAb was interacting with its target (43). However, solanezumab failed to meet the primary clinical endpoints in three Phase 3 treatment trials and in a secondary prevention trial (Dominantly Inherited Alzheimer Network Trial: DIAN) that treated asymptomatic or mildly symptomatic individuals carrying AD-pathogenic mutations (44, 45). Two of the Phase 3 studies discovered that a high proportion of study subjects did not have high initial levels of amyloid in their brains; this has led to the addition of positive amyloid PET or pathological CSF biomarkers in the inclusion criteria for more recent clinical studies. Subsequent pooled analyses of trial data revealed a slowing of cognitive decline in individuals with mild AD (44), and an open-label extension study in patients who had completed Phase 3 trials suggested that solanezumab produced a cognitive benefit consistent with a treatment effect on the underlying AD pathology (46). Solanezumab has been tested in asymptomatic or very mildly symptomatic people with biomarker evidence of brain amyloid deposition as part of the Anti-Amyloid Treatment in Alzheimer’s Disease Prevention Trial (A4) but did not slow cognitive decline or reduce progression to symptomatic AD (47).

Crenezumab (Genentech/Roche) is a humanized mAb that binds oligomeric and insoluble fibrillar forms of Aβ while also binding to monomeric Aβ. The initial results of Phase 3 CREAD trials of crenezumab in prodromal or mild AD reported that although it was well-tolerated, there were no effects on the primary or secondary outcomes (48). This mAb is also included in the Alzheimer’s Prevention Initiative (API) trial, where its effects on cognition and biomarkers are being evaluated in presymptomatic carriers of the autosomal-dominant PSEN1 E280A mutation. The results of this study have been negative, despite some positive trends toward slowing declines in primary and secondary outcomes relating to cognition, biomarkers, and neuropathology.

Gantenerumab (Roche) is a fully human mAb that targets N-terminal and mid-regions of Aβ and preferentially binds to aggregated fibrillar Aβ (49). Phase 2/3 trials in subjects with prodromal or mild AD who were positive for amyloid PET reported no efficacy on primary or secondary endpoints (SCarlet RoAD) or failed an interim futility analysis (Marguerite RoAD) (50). The incidence of ARIA-E was also fairly high (30%) and correlated with dose and the number of ApoE4 alleles carried by the patient. Two- and three-year open label extensions of these studies using a higher dose of gantenerumab found ongoing reductions in brain amyloid assessed using florbetapir PET; around one-third of participants developed ARIA-E, although the majority were asymptomatic (51, 52). Gantenerumab treatment also increased plasma Aβ40 and Aβ42 and decreased phosphorylated tau levels as well as slowing cognitive decline. The DIAN trial of this mAb missed its primary endpoint, but some positive benefits were noted, and an open-label extension using high-dose gantenerumab for up to 3 years started in late 2020. Additional two Phase 3 trials of subcutaneous gantenerumab or placebo in prodromal or mild amyloid-confirmed AD (GRADUATE 1 and 2) completed in 2022, with a disappointing press release (November 2022), indicating that they had failed to meet their primary endpoints of slowing clinical decline (53). Although Roche began a Phase 3 secondary prevention trial of gantenerumab (SKYLINE) in March 2022, they have since discontinued this study. A phase 2 study is ongoing with trontinemab (gantenerumab coupled with a brain delivery technology).

Aducanumab (Biogen/Neurimmune/Eisai) is a fully human mAb that shows strong binding to aggregated Aβ fibrils and has a low affinity for Aβ monomers. Two Phase 3 trials (ENGAGE and EMERGE) were initiated in 2015 in subjects with mild cognitive impairment (MCI) due to AD or mild AD with positive amyloid PET scans. Despite terminating these trials in March 2019 because interim analyses indicated that they would miss their primary endpoints, Biogen reported an analysis of a larger dataset later that year, showing that EMERGE had met its primary cognitive endpoint and some secondary endpoints in people taking the highest dose. These data showed profound plaque clearance and an efficacy signal supporting progression of the clinical program (54–57). Approximately one-third of participants administered aducanumab-developed ARIA, with 3% showing serious symptoms. Those who had brain microhemorrhages at baseline or carriers of ApoE4 were at greater risk for this adverse event (58). A Phase 3b open-label study for 2,400 previous aducanumab trial participants (EMBARK) will run until late 2023. Biogen has applied for approval for clinical use of aducanumab in multiple territories under the trade name of Aduhelm. Although approved by the US Food and Drug Administration in 2021 via the accelerated approval pathway, this was a controversial decision because of the weaknesses of the Phase 3 data, and Aduhelm has not been approved by the European Medicines Agency.

Donanemab is a humanized mAb that recognizes an N-terminally truncated and pyroglutamate-modified form of Aβ that is found aggregated in amyloid plaque cores. Lilly embarked on a series of Phase 2 and Phase 3 TRAILBLAZER trials of donanemab in 2017. Positive top-line TRAILBLAZER-ALZ 2 phase 3 results were reported by Lilly in May 2023. TRAILBLAZER-ALZ 2, which enrolled patients with early cognitive decline and positive amyloid PET scans, found that donanemab slowed cognitive decline (measured by the integrated AD rating scale) by 22% as compared to placebo at 18 months, reduced amyloid plaque load, and slowed the rate of cortical neurofibrillary tangle accumulation. ARIA-E developed in 24% of treated patients (6% symptomatic). This adverse event was classed as serious in 1.6% of treated patients (59). Larger scale TRAILBLAZER-ALZ 3, 4, and 5 trials are ongoing in patients with early AD or in those who are cognitively normal, to explore disease prevention. Although the FDA rejected Lilly’s accelerated approval application for donanemab in January 2023 due to insufficient safety data, the company has since then applied for full approval using the TRAILBLAZER-ALZ 2 results.

For further information about the current progress of potential therapies for AD, AlzForum (60) provides an excellent online resource.

## The development of a novel therapeutic antibody targeting Aβ protofibrils

### Lecanemab development

In parallel with the development of the mAbs described earlier, our research group at Uppsala University had identified soluble Aβ aggregates (oligomers and protofibrils) as a viable therapeutic target for AD. This developed from our identification of the Arctic Aβ mutation and the observations that the resultant Aβ peptide formed large soluble Aβ protofibrils (18–20) and that individuals with the mutation lacked aggregated fibrillar amyloid (25), taken together with previous work showing that soluble Aβ species in the 75–500 kDa size range were neurotoxic (15). We generated a murine mAb (mAb158) with a binding profile that was conformation-dependent, rather than targeted to a linear epitope of Aβ. This mAb selectively bound to soluble oligomeric forms of Aβ, with low affinity for either monomers or higher order insoluble fibrillar Aβ aggregates (61–63). We treated young (4 months old) and elderly (14 months old) ArcSwe transgenic mice with mAb158. No effect was observed on the levels of insoluble Aβ in the brains of older mice that had already developed amyloid plaques. However, the antibody prevented plaque formation in the younger mice, who had yet to develop plaque pathology. In both cases, soluble Aβ protofibril levels decreased (64). This demonstrated that mAb158 selectively reduced protofibril levels *in vivo*.

**Figure 3:**
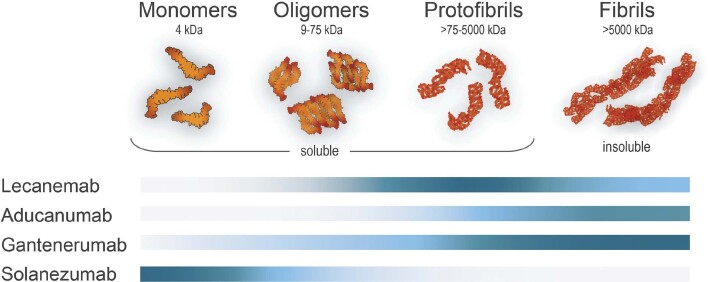
Schematic illustration of the Aβ binding profiles of the indicated therapeutic antibodies evaluated in clinical trials, where a darker blue color indicates stronger binding affinity. For example, lecanemab targets preferentially the neurotoxic soluble oligomeric and protofibrillar forms of Aβ. This illustration is based on data reported by Söderberg et al., 2022 (65).

A humanized version of mAb158 known as BAN2401 was then developed by BioArctic, with binding characteristics that were essentially indistinguishable from those of mAb158. A licensing agreement with Eisai in 2007 led to further development of BAN2401, known as lecanemab. Consistent with earlier studies of mAb158 (62), *in vitro* analyses have shown a >2000-fold and 10-fold selectivity of lecanemab for protofibrils over Aβ monomers or fibrils, respectively (Figure 3) (65). This contrasted with aducanumab and gantenerumab, which demonstrated selectivity for fibrils over protofibrils (65).

### Lecanemab trials

A Phase 2 clinical study was conducted in patients with either early-stage AD or MCI due to AD, with a positive amyloid PET scan. The highest dose (10 mg/kg, twice-monthly) resulted in up to 93% of patients being amyloid plaque negative and slowed cognitive decline by 26–47%, depending on the assessment scale employed (66). Magnetic resonance imaging detected ARIA-E in around 10% of participants overall, and in <15% of those with ApoE4 in the highest-dose group; most of these cases were asymptomatic.

Based on these promising Phase 2 data, the Clarity AD Phase 3 trial was initiated in March 2019. This trial treated 1,795 individuals with early symptomatic AD with 10 mg/kg lecanemab (*n* = 898) or placebo (*n* = 897) twice-monthly for 18 months, followed by a 2-year open label extension. Lecanemab slowed decline on the primary Clinical Dementia Rating endpoint by 27% over 18 months and also slowed decline on all the key secondary clinical endpoints (67). Furthermore, lecanemab demonstrated a clear effect on important biomarkers such as phosphorylated tau and neurofilament light. ARIA-E was observed in 12.6% of treated patients. A sub-study of 698 participants showed a large reduction in brain amyloid burden with lecanemab compared to a slight increase with placebo. Two other clinical trials of lecanemab are ongoing: a large Phase 3 trial (AHEAD 3-45) in people who are cognitively normal but have elevated brain amyloid, and the first DIAN-TU prevention study of concurrent therapies targeting amyloid and tau in people with familial AD mutations, pairing lecanemab with an anti-tau humanized mAb known as E2814 (Eisai) (68). A subcutaneous form of lecanemab is under evaluation, which would be a more convenient way of administrating for the patients (Figure 2).

In July 2023, the FDA gave lecanemab a full approval, and this therapeutic mAb marketed as Leqembi® in the USA.

## Clinical promise and limitations of Aβ immunotherapy

Although many of the attempts to develop immunotherapies for AD to date have ended in a failure to demonstrate positive benefits in clinical trials, some recent studies have provided clear evidence that passive Aβ immunotherapy can have positive effects. It is difficult to assess and compare the efficacy and safety of these potential therapies because of differences in trial design, study populations, dementia rating scales, and endpoints. AD clinical trial methodology that was developed for small-molecule drugs during the 1980s and 1990s has had to adapt in several ways to incorporate an improved understanding of the disease process and the new classes of potential therapeutic agents. Difficulties with the clinical diagnosis of AD mean that a subset of each clinical trial population may be misdiagnosed; this issue affected the bapineuzumab trials and a significant proportion of patients included in the solanezumab trials turned out to lack high levels of brain amyloid deposits. The more recent immunotherapy trials have, therefore, used additional inclusion and exclusion criteria to improve diagnostic accuracy, including positive detection of Aβ or tau pathology consistent with AD using PET neuroimaging. Amyloid PET imaging is also being evaluated in clinical trials as a potential marker of disease progression. In addition, it is important to include appropriate baseline genetic and biochemical analyses. The measurement of CSF biomarkers such as various forms of Aβ and tau can provide support for diagnosis and act as biomarkers of disease progression (69). However, there is a lack of consensus about the direction and magnitude of change in a particular biomarker that is necessary to predict a clinical effect. For example, depending on the agent under study and the assay methods employed, plasma and CSF Aβ levels may increase, decrease, or stay the same after treatment. A more rigorous understanding of the mechanisms underlying these changes in biomarker levels is required to interpret these trial read-outs more accurately. To this end, the A4, API, and DIAN longitudinal clinical trials mentioned earlier include investigations of pre-clinical changes in AD, with the aim of obtaining regulatory support for the validity of biomarkers for both diagnosis and monitoring of disease progression.

Attrition is another important issue for the validity of AD immunotherapy trials. Past trials of symptomatic treatments lasted only 3–6 months and typically experienced attrition rates of 10–15%. However, immunotherapy trials that aim to detect potential disease-modifying effects are typically 18–24 months in duration and can have attrition rates as high as 20–40%. Shorter duration trials that achieve similar goals without high attrition may become possible with increased use of biomarker and imaging outcomes, if the regulatory environment becomes more open to approving drugs based on biomarker findings rather than clinical measures.

The experience gained from previous immunotherapy trials has highlighted the importance of targeting the very early stages of the disease process, which begin many years before the onset of symptoms (6, 70). Although the patients included in clinical trials have traditionally been diagnosed with mild to moderate AD, targeting Aβ at this stage of the disease might be too late. Several studies have found elevated levels of soluble Aβ very early in the disease, and this change is likely to precede clinical symptoms (71). The ideal population for immunotherapy treatment is, therefore, presymptomatic individuals or patients with MCI associated with amyloid neuropathology. It is possible that the severity of disease in some previous trial cohorts did not allow for clinical improvement because the treatment was provided too late in the disease process. When measuring treatment effects, it is also important to employ cognitive assessments that are sensitive enough to detect meaningful changes. The methods traditionally used to analyze cognitive outcomes often lack sensitivity in patients with early AD or MCI. Eisai has developed a more sensitive Alzheimer Disease Composite Score (ADCOMS) derived from existing Mini-Mental State Exam and Clinical Dementia Rating instruments to improve diagnosis and tracking of treatment effects in clinical trials (72).

The major side-effect associated with AD immunotherapy is ARIA (mainly ARIA-E), which tends to occur at an early stage of treatment. The incidence of ARIA-E is greater in patients receiving higher antibody doses and in those carrying ApoE4 (73). The exact mechanism underlying this adverse event has not yet been elucidated, but it may relate to therapeutic mAbs binding to CAA (74, 75). This could release Aβ and trigger a local inflammatory reaction leading to impairment of the BBB and subsequent edema (76, 77). Because CAA consists of fibrillar Aβ 1‑40 (78), mAbs with a low affinity for this Aβ species (such as solanezumab and lecanemab; Figure 3) are theoretically less likely to cause ARIA-E. This has been supported by clinical data, where ARIA-E has been observed with the following frequencies: not significantly higher than placebo for solanezumab (binds soluble monomeric Aβ) (79); 12.6% for lecanemab (binds Aβ protofibrils/oligomers) (67), 35% for aducanumab (binds Aβ fibrils) (54–57), 30% for gantenerumab (binds Aβ fibrils) (56), and 24% for donanemab (binds amyloid plaques) (59).

Currently available data indicate that both lecanemab and donanemab significantly slow cognitive and functional decline in patients with early symptomatic AD by around 30‑40% over 18 months. In relation to adverse events, the proportion of patients who developed ARIA-E was about twofold higher following treatment with donanemab, as compared with lecanemab (67, 80). However, the differences in study design discussed above make it difficult to compare the relative efficacies of these two mAbs precisely. For example, the inclusion criteria for the TRAILBLAZER-ALZ 2 study included tau-PET pathology, and the participants were older and had lower mini mental state examination scores (by approximately 3 points) than those included in the lecanemab Phase 3 study. In addition, the stratified TRAILBLAZER-ALZ 2 data analysis focused on a subpopulation with intermediate tau-PET pathology, while the lecanemab Phase 3 study included patients with a wider range of disease severity. These differences render direct comparisons of the Clinical Dementia Rating Scale Sum of Boxes data unreliable, even though this cognitive scale was used in both studies. Despite these difficulties in determining the relative efficacy of these two different mAbs, the findings reported to date are promising.

## Future perspectives and challenges

The past four decades of research into the pathogenesis of AD have identified many valid therapeutic targets within the slow cascade of molecular events that develop over many years and ultimately result in the devastating cognitive deficits of AD. The frequent setbacks littering the clinical development pathways of anti-Aβ small molecules and immunotherapies have understandably undermined the conviction that Aβ is the correct target to pursue. However, multiple lines of research evidence support the idea that a change in Aβ production, aggregation state, or removal/degradation is sufficient to initiate this pathological cascade, indicating that this molecule acts very early in the disease process. If the downstream events in the cascade triggered by Aβ are irreversible once initiated, this could explain why many of the anti-Aβ agents tested in humans have been proven to interact with their target, as shown by biomarker effects, without producing convincing clinical improvement. If correct, this view of the disease dictates that any therapy targeting Aβ should ideally be deployed in individuals who will develop AD in the future but who do not yet have any clinical symptoms; this is problematic because there are not yet any approved diagnostic biomarkers that are sensitive and specific enough to identify these people. Studies aimed at developing appropriate methods to assess an individual’s risk for future AD will, therefore, be crucial for the effective testing and application of immunotherapies targeting Aβ.

It is important to remember that while there is unquestionably a major need for AD prevention initiatives, abandoning attempts to develop new treatments for those already suffering from dementia could have a disastrous effect on prevalence in the next few decades as large proportions of global populations grow older. The development of therapies targeting molecules that are downstream of Aβ in the pathogenesis of AD and improving the design of clinical studies involving symptomatic patients with AD, therefore, remain important goals.

The preclinical and clinical data relating to mAb158 and lecanemab demonstrate that this mAb preferentially targets soluble protofibrils, a neurotoxic form of Aβ. The use of improved imaging, biochemical and cognitive instruments for diagnosis, and treatment effect monitoring in clinical trials has produced very promising data, indicating that lecanemab can slow the progression of AD, with a low incidence of adverse events. Despite all the set-backs, there is, therefore, considerable cause for optimism that lecanemab and other immunotherapeutic agents can offer new hope for innovative approaches to the prevention and/or treatment of AD (81).
